# Use of Antiangiogenic Therapies in Pediatric Solid Tumors

**DOI:** 10.3390/cancers13020253

**Published:** 2021-01-12

**Authors:** Claudia Ollauri-Ibáñez, Itziar Astigarraga

**Affiliations:** 1 Pediatric Oncology Group, BioCruces Bizkaia Health Research Institute, 48903 Barakaldo, Spain; collauri.osaki@gmail.com; 2 Pediatrics Department, Hospital Universitario Cruces, 48903 Barakaldo, Spain; 3 Pediatrics Department, University of the Basque Country UPV/EHU, 48940 Leioa, Spain

**Keywords:** cancer, solid tumors, pediatric, childhood, angiogenesis, antiangiogenic drugs, therapy

## Abstract

**Simple Summary:**

Cancer is one of the leading causes of death in children worldwide. One of the basic mechanisms for cancer development, both in children and in adults, is angiogenesis. This made it an important target for the development of antitumor drugs. Unfortunately, the clinical benefits of drugs that inhibit this process have been much less than initially expected. This review summarizes what are the main antiangiogenic drugs, against which signaling pathways they are targeted, and the results that have been obtained from their use in pediatric patients. It also includes the basic information of the clinical trials that are currently underway using antiangiogenics and that include children.

**Abstract:**

Cancer is an important cause of death in childhood. In recent years, scientists have made an important effort to achieve greater precision and more personalized treatments against cancer. But since only a few pediatric patients have identifiable therapeutic targets, other ways to stop the neoplastic cell proliferation and dissemination are needed. Therefore, the inhibition of general processes involved in the growth and behavior of tumors can be a relevant strategy for the development of new cancer therapies. In the case of solid tumors, one of these processes is angiogenesis, essential for tumor growth and generation of metastases. This review summarizes the results obtained with the use of antiangiogenic drugs in the main pediatric malignant solid tumors and also an overview of clinical trials currently underway. It should be noted that due to the rarity and heterogeneity of the different types of pediatric cancer, most studies on antiangiogenic drugs include only a small number of patients or isolated clinical cases, so they are not conclusive and further studies are needed.

## 1. Introduction

Cancer is one of the leading causes of death among children and adolescents [1]. However, in absolute numbers, childhood cancer is a rare disease with a five-year survival rate around 80% in high-income countries and 40% in low- and middle-income countries [2,3]. This survival has improved for leukemias and lymphomas, but plateaued for many solid tumors.

The number of anticancer therapies approved for childhood cancer is significantly lower than for adults because they have special requirements, such as generating longer-term results and fewer side effects [4], and the clinical trials are difficult due to the low prevalence. So, between 1980 and 2017, the Food and Drug Administration (FDA) approved only 11 antitumor drugs for children [4].

### Origin of Childhood Cancer

Although the pathogenesis of pediatric cancer is unknown, the impact of nongenetic factors is relevant. This way, hereditary cancers like retinoblastoma or predisposing cancer syndromes are only 5–10% of childhood malignancies [5,6,7,8].

Some pediatric tumors appear to be related to upregulation and downregulation of gene or protein expression, but few of them express an identifiable therapeutic target [9,10]. Therefore, although the goal of cancer therapy remains precision medicine and personalized treatment based on tumor-specific alterations, nowadays the benefits in pediatric cancer are minimal. For this reason, more general mechanisms cannot yet be ruled out for the development of new drugs. In the case of solid tumors, one of these mechanisms is angiogenesis.

## 2. Tumor Angiogenesis

Angiogenesis is the process by which new blood vessels are formed by sprouting from pre-existing ones. It is involved in numerous pathophysiological processes, among which cancer stands out [11,12,13]. The angiogenic process includes the production of proteases that degrade the extracellular matrix (ECM), selection and migration of tip endothelial cells (ECs) toward the angiogenic stimulus, proliferation of stalk ECs, lumen formation, anastomosis of newly formed vessels, synthesis of a new basement membrane, and incorporation of mural cells (pericytes and vascular smooth muscle cells) [11,14,15,16].

In the case of tumors, rapid cell proliferation, with the consequent growth of malignant tissue, makes the demand for nutrients and oxygen and the need for waste removal very high [17]. Tumor cells secrete proangiogenic molecules that bind to their receptors in the ECs of the nearby blood vessels and initiate the formation of new vessels through a process very similar to physiological angiogenesis, but which results in a disordered, defective, and deformed vasculature as a consequence of the high concentration of proangiogenic factors [16,18,19,20].

Angiogenesis has another fundamental role in the development of cancer: the generation of metastases. The leakiness and high permeability of the tumor vessels allow the extravasation of blood into the tumor stroma but also the intravasation of the tumor cells, which travel in the blood until they colonize other locations and create secondary tumors [18,20].

### Main Targets of Antiangiogenic Therapy

Since Dr. Folkman discovered that a tumor cannot grow more than few millimeters without the presence of blood vessels [17], many attempts have been made to block this mechanism to deprive tumor cells of “nutrition” and avoid the generation of metastases.

**Figure 1:**
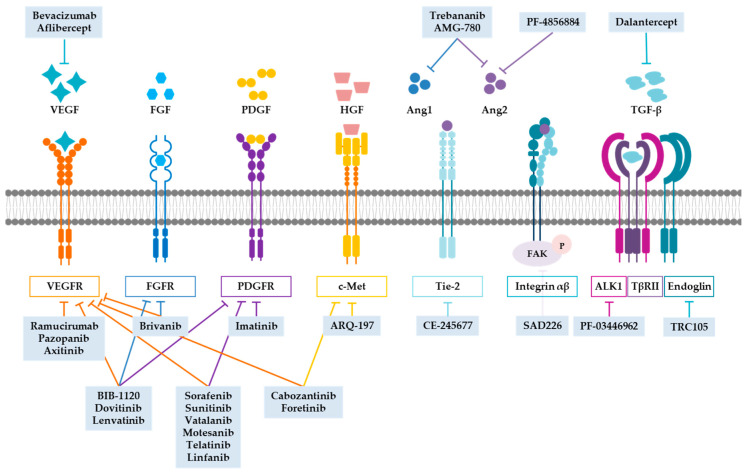
Main target signaling pathways of antiangiogenics. VEGF was the first signaling pathway inhibited by antiangiogenic drugs. Unfortunately, the benefits of anti-VEGF/VEGFR are limited and patients develop resistance. Therefore, drugs were developed that inhibit alternative signaling pathways such as FGF and PDGF. Multikinase inhibitors (MKIs) simultaneously target different components of these pathways. HGF and its receptor c-Met can also be inhibited by antiangiogenics. The angiopoietin pathway can be inhibited by preventing it traditional signaling through Tie2 or through integrins. There are also antiangiogenic drugs against TGF-β and its receptors ALK1 and endoglin.

Vascular endothelial growth factors (VEGFs), and more specifically VEGF-A, are the most important proangiogenic stimuli. VEGF activates cell signaling by binding to VEGF receptor (VEGFR), stimulating the proliferation and survival of ECs and increasing vessel permeability [13,16]. The first antiangiogenic drug was bevacizumab, a humanized monoclonal antibody that binds to all circulating forms of VEGF-A, blocking its binding to VEGFRs. It was approved by the FDA in 2004 and by the European Medicines Agency (EMA) in 2005 for the treatment of metastatic colorectal cancer, but is currently used in other types of tumors, including lung, breast, kidney, ovarian, cervical, or glioblastoma [13,21]. In addition to bevacizumab, there are other drugs that inhibit the binding of VEGF to its receptors (aflibercept) and antibodies or inhibitors that block signaling through VEGFR (ramucirumab, pazopanib, and axitinib) [21] (Figure 1).

Although initially VEGF inhibitors were promising, it was soon seen that their clinical efficacy was limited and produced upregulation of other proangiogenic signals, toxicity, and other side effects [13,22]. For this reason, it was proposed that combining drugs that inhibit VEGF/VEGFR with drugs that block alternative pathways could avoid resistances and increase the effectiveness. Alternative pathways against which drugs have been tested with promising results include platelet-derived growth factor (PDGF), fibroblast growth factor (FGF), hepatocyte growth factor (HGF), angiopoietins, and transforming growth factor β (TGF-β) (Figure 1), among others.

PDGF and its receptors (PDGFRs) are involved in mural cell recruitment and vessel stabilization. During the final phases of angiogenesis, ECs of the new vessels release PDGF-B, which attracts pericytes expressing PDGFR-β [23,24,25]. Overexpression of PDGF blocks angiogenesis by inhibiting EC proliferation and increases pericyte recruitment in vivo [24,26]. However, we have not found studies on systemic administration of PDGF in patients. The PDGFR prototype inhibitor, imatinib, which also inhibits c-kit and c-Abl, has become the standard treatment for chronic myeloid leukemia (CML) and gastrointestinal stromal tumor (GIST) with excellent results. Imatinib use is extending to other cancers such as Ph+ acute lymphoblastic leukemia (ALL), glioma, or prostate cancer [27,28]. Moreover, PDGFR inhibition seems to be really efficient when combined with VEGF/VEGFR inhibitors or cytotoxic drugs [24].

FGF family plays many roles in normal and tumor cells. It has been shown to stimulate angiogenesis through increasing other proangiogenic factors [29]. Blocking FGFR and its downstream signaling pathway results in vessel disintegration and inhibition of EC proliferation and enhances the efficacy of VEGFR and PDGFR inhibitors [24]. There is currently no approved FGFR therapy for cancer treatment, but many clinical trials are underway with monoclonal antibodies against FGFR, FGF ligand traps, and FGFR kinase domain inhibitors [30,31].

Although there is still some controversy about their usefulness, several multikinase inhibitors (MKIs) have been developed that target VEGFRs, PDGFRs, FGFRs, and their signaling pathways. Among these MKIs, some of them target VEGFRs and FGFRs (brivanib), some VEGFRs and PDGFRs (sorafenib, sunitinib, vatalanib, motesanib, telatinib, linfanib), and others VEGFRs, FGFRs, and PDGFRs (BIB-1120, CHIR-258, lenvatinib) [24].

Another factor that has gained importance in recent years is HGF, although it is not one of the traditional routes of angiogenesis. HGF binds to its receptor c-Met and activates the Src, PI3K/Akt, and Ras/MEK pathways. It stimulates EC proliferation and the release of proangiogenic factors [32]. c-Met inhibitors ARQ-197 and c-Met and VEGFR inhibitors cabozantinib and foretinib have demonstrated antitumor activity [24].

The angiopoietin family, especially Ang1 and Ang2, and its receptor Tie2 play an important role in angiogenesis. Although Ang1 and Ang2 have a great homology, they have antagonistic effects. While Ang1 promotes the stabilization of mature vessels, Ang2 stimulates vascular destabilization and abnormal EC proliferation [16,24,33,34]. A decrease in the Ang1/Ang2 ratio has been observed in some malignancies [33,34], favoring the creation of leaky vessels. All this has led to the development of Ang2 inhibitors (PF-4856884) and Tie2 inhibitors (CE-245677) [24]. The role of Ang1 in tumor angiogenesis is unclear, since its overexpression seems to have either proangiogenic and protumor effect or antiangiogenic and antitumor effect depending on the specific type of cancer [35]. In any case, the results of simultaneously inhibiting Ang1 and Ang2 with inhibitors such as trebananib and AMG-780 are promising [24,34,36].

In addition to Ang2/Tie2 signaling, Ang2 can also promote angiogenesis by binding to integrins αvβ3, αvβ5, and α5β1. This binding stimulates the phosphorylation of focal adhesion kinase (FAK), promoting EC migration and aberrant sprouting [33,34]. Some integrin inhibitors have been shown to reduce angiogenesis and tumor growth. However, when they reached the clinic, such as etaracizumab, they had no antiangiogenic effect or even increased this process [37]. Attempts have also been made to block FAK. Some inhibitors are able to suppress tumor angiogenesis, such as SAD226, although these are still in preclinical or early clinical phases [38,39].

The TGF-β family is involved in different physiological processes including wound healing, immune response, and angiogenesis. TGF-β acts as a tumor suppressor in normal and premalignant tissues, but favors metastasis and angiogenesis in neoplastic tissue [40,41]. In the ECs, the TGF-β receptor complex is formed by TβRI (ALK1 or ALK5), TβRII, and endoglin. In the conventional TGF-β signaling pathway, ALK5 signals through Smad2/3, inhibiting EC proliferation and migration, while ALK1, which signals through Smad1/5/8, promotes angiogenesis [11,12,42]. ALK1 overexpression has been observed in different types of solid tumors [42,43,44]. PF-03446962 is a humanized monoclonal antibody against ALK1 with promising results in preclinical and early clinical trials [21,42]. Another way to block TGF-β signaling through ALK1 is dalantercept, a soluble protein fusion formed by the extracellular domain of ALK1 linked to IgG1-Fc and which binds to some members of the TGF-β family, preventing its binding to the receptor complex [42]. Endoglin (CD105) is an auxiliary receptor of TGF-β that promotes ALK1 signaling [11,12]. It is considered a proangiogenic molecule, since its expression increases in the angiogenic front and its overexpression in microvessels is related to a poor prognosis in different cancers [11,12,45]. Moreover, its deficiency produces alterations in vessel structure and inhibits tumor growth [11,12,45]. Recently it has been demonstrated that continuous overexpression of endoglin prevents vessel maturation and facilitates the generation of metastases [45]. TRC105 is a monoclonal anti-endoglin antibody that has shown good results in inhibiting tumor growth and metastasis [12,46,47].

Other drugs are out of the scope of this review because they do not have a specific target but they have antiangiogenic properties, like thalidomide and derivatives [16,48], interferons [16], and metronomic chemotherapy. This type of treatment consists of frequent or continuous administration of chemotherapeutic agents at low doses, which is less toxic than conventional [49]. It targets the tumor microenvironment (TME) by inhibiting angiogenesis and stimulating antitumor immune response.

Many of the mentioned antiangiogenic drugs have been proven in pediatric patients or in preclinical models of tumors that frequently appear in childhood. However, most studies evaluate the effect against tumor cells and not specifically their antiangiogenic capacity. Moreover, few clinical trials with antiangiogenic drugs have been developed for pediatric cancer, therefore the results are scarce.

## 3. Antiangiogenics and Brain Tumors

Primary tumors of the central nervous system (CNS) are the most common malignancies in children after leukemia [50], and include gliomas like astrocytomas, and embryonal tumors, including medulloblastoma. Survival depends on many factors like tumor type, location, age, and resectability.

Benefits of antiangiogenic therapy in brain tumors are not clear. It is usually well tolerated in children, but the tumor response is poor [16]. By contrast, bevacizumab has been successfully used to treat radiation-induced necrosis in several brain tumors [51,52,53].

### 3.1. Low-Grade Gliomas

Low-grade gliomas are the most common pediatric brain tumor. Since some of these tumors like pilocytic astrocytoma are highly vascularized [54,55], antiangiogenics may be recommended. In fact, bevacizumab as a single agent reduces tumor volume and apparent diffusion coefficient [16,56,57], and improves field of vision, cranial neuropathy, strength, and gait without tumor progression in some cases [58]. The combination of bevacizumab with conventional chemotherapy is well tolerated and more effective in preserving vision and improving mobility in patients with inoperable or progressive low-grade glioma [59]. It also helps to preserve vision in patients with optic pathway gliomas in combination with carboplatin or proton-beam radiation [60]. In some patients treated with bevacizumab and irinotecan, a stabilization or even a tumor reduction and clinical improvements were observed [61,62,63,64,65]. In general, toxicity is moderated, but cases of severe vascular stenosis and tumor hemorrhage have been described [66]. Also, the administration of bevacizumab with dabrafenib and trametinib in astrocytomas has improved the quality of life [67]. Some patients progressed at the end of treatment with bevacizumab, but the retreatment controlled the disease again [57,62,68].

Sorafenib has also been tested, but it produced unexpected unprecedented tumor growth in patients with progressive low-grade astrocytomas [69,70]. In addition, ascites can appear in patients with ventricular-peritoneal shunt and it disappears when treatment is discontinued [70].

### 3.2. Medulloblastomas

Medulloblastomas are embryonal tumors [50] and the second most frequent brain tumors in childhood [16]. The role of angiogenesis in these tumors is not well characterized, but it seems to be especially important in group 3, which has the worst prognosis [71].

Combination of bevacizumab with other drugs in metronomic chemotherapy (thalidomide, celecoxib, fenofibrate, etoposide, cyclophosphamide, and liposomal cytarabine) has benefits and increases progression-free survival (PFS) [16,72], so a clinical trial (MEMMAT) is recruiting patients nowadays. The combination of bevacizumab with irinotecan, with or without temozolomide, in relapsed medulloblastomas, and with tensirolimus in chemoresistant medulloblastomas has shown some effects in clinical reports [63,73,74].

In preclinical models, sunitinib and pazopanib have been shown to reduce the proliferation and migration of medulloblastoma cell lines [75,76,77]. Pazopanib also inhibits intracranial tumor growth and increases survival in animal models [75].

### 3.3. High-Grade Gliomas

High-grade gliomas include anaplastic astrocytoma, multiform glioblastoma, diffuse midline glioma, and diffuse intrinsic pontine glioma (DIPG). High-grade gliomas are generally considered to have a high microvascular density (MVD) [78]. They express high levels of VEGF [54] and PDGFRα [79], so these pathways are potential therapeutic targets.

Bevacizumab increases overall survival (OS) and PFS in adult patients [54], but in children the response rates and PFS are lower [16,80,81,82]. Many regimens combine bevacizumab and chemotherapy. The combination with irinotecan produces a moderate toxicity, but the impact in survival is variable or not effective in different studies [54,63,80,82,83]. Bevacizumab is also administrated with temozolomide and radiation with variable results [84,85]. This combination was also used in the phase II trial HERBY. In this study, 59 newly diagnosed high-grade glioma patients were treated with radiation and temozolomide, and another 62 patients received the same regimen and bevacizumab. Results showed that the addition of this VEGF-inhibitor did not improve PFS and OS, but increased side effects [86]. Bevacizumab administration with tensirolimus is also well tolerated and generates partial response or stabilization in high-grade gliomas [74], but the combination of the antiangiogenic with valproic acid does not improve PFS or OS [87].

On the other hand, administration of sorafenib and valproic acid reduced tumor size and clinical symptoms in a child with spinal glioblastoma [88], while sunitinib did not produce sustained tumor response in patients with high-grade glioma [89].

Other VEGF/VEGFR inhibitors that have been tested in high-grade glioma preclinical models are cabozantinib, which reduces proliferation and migration and induces apoptosis in DIPG cell lines [90]; foretinib, which inhibits tumor growth in animal models of glioblastoma [91]; and aflibercept, which increases Ang2 expression in glioblastoma murine models, but in combination with trebananib increases survival, reduces permeability and the amount of tumor-associated macrophages, improves mural cell coverage, and increases lymphocyte infiltration [92]. Trebananib was tested in a phase I clinical trial in children with relapsed solid tumors, being well tolerated but with little response, except two long-term stabilizations, one of them in anaplastic astrocytoma [93].

### 3.4. Ependymomas

Ependymomas are tumors created from ependymal cells lining the ventricles of the brain and the spinal cord. In this type of cancer, bevacizumab appears to have a minimal efficacy in most studies [16,94,95]. The combination of bevacizumab and irinotecan reduces mean diffusion ratio, but it does not correlate with PFS and fails to stop progression in patients with recurrent or refractory ependymoma [65,94]. Bevacizumab with chemotherapy also fails to stop progression of a second tumor in a child with supratentorial ependymoma associated with Li-Fraumeni syndrome [96]. On the contrary, the combination of bevacizumab and tensirolimus produced stabilization or partial remission in chemoresistant ependymomas [74].

Another VEGF pathway inhibitor, pazopanib, was shown in a phase I study to stabilize the tumor in a patient diagnosed with ependymoma for more than six months [97]. Cabozantinib also stabilized ependymoma in one case [98], but no sustained tumor response was observed in recurrent or refractory patients treated with sunitinib [89].

In preclinical ependymoma assays, axitinib, imatinib, and pazopanib have shown promising results [99], so they may soon jump into clinical trials.

### 3.5. Other Brain Tumors

Antiangiogenics has been used in other less common brain tumors. Results obtained with bevacizumab and sorafenib are contradictory and no recommendation about its use can be established [100,101,102]. Sunitinib combined with sirolimus, thalidomide, and vorinotad reduced choroid plexus carcinoma size without side effects in one case report [103].

Despite brain tumors having a high expression of PDGFR, imatinib’s efficacy is disappointing. A phase I study showed that it increases the risk of intratumoral bleeding and a phase II study saw no response. One possible explanation is that only 5% of imatinib’s plasma levels cross the blood–brain barrier [16].

## 4. Antiangiogenics and Neuroblastoma

Over 15% of childhood cancer deaths are due to neuroblastoma, an extremely heterogeneous tumor that arises in the neural crest. Neuroblastomas are divided into low-, intermediate-, and high-risk groups based on histology, clinical status, tumor cell ploidy, and MYCN oncogene amplification. Survival varies from nearly 100% for low-risk patients to less than 40% in the high-risk group. Their current treatment combines surgery, chemotherapy, radiation, and immunotherapy [104].

High-risk neuroblastomas are characterized by being very aggressive and have a highly vascular, angiogenic, hypoxic, and immunosuppressive TME [104,105,106]. They also express large amounts of VEGF, PlGF, FGF, PDGF, and endoglin [104,107,108,109,110,111], so some antiangiogenics against these targets have been used in patients and preclinical models of neuroblastoma.

Most studies on bevacizumab treatment have developed in xenografts. Bevacizumab has been shown to inhibit the tumor growth [105,112,113], normalize vessels, and improve the arrival and efficacy of chemotherapy [105,113], especially if administrated three days after bevacizumab [114]. Surprisingly, VEGF and FGF expression increases during bevacizumab treatment, which could lead to resistance [115]. Other studies did not see much efficacy of bevacizumab as monotherapy, but it increased when HIF-1α expression was inhibited with shRNA, topotecan, or nutlin-3a [116,117]. The combination of bevacizumab and the Trk inhibitor lestaurtinib prevents tumor growth dramatically, but with significant toxicity [118]. Bevacizumab also increases antitumor activity of GD2-CAR T cells [119] and has been used to target doxorubicin-loaded nanoparticles (SiO_2_@LDH-Bev) to the tumor, avoiding doxorubicin effects on healthy tissues [120]. In patients, some partial responses have been observed, but also toxicity like bowel perforation [121].

Ramucirumab inhibits cord formation by neuroblastoma cells and its analog DC101 delays or regresses xenograft growth, decreases vasculature, and increases chemotherapy effectiveness [105,122]. Pazopanib does not reduce cell viability as a single agent, but it does when combine with topotecan [123]. This combination is also effective in xenograft models [123,124], although long-term treatment increases mural cell coverage and resistance to treatment [124]. Axitinib also reduces proliferation and colony formation of neuroblastoma cells, delays growth, and reduces size and MVD in xenografts [125,126].

Although sunitinib has low antitumor activity in neuroblastoma cells according to the pediatric preclinical testing program (PPTP) [127], both sunitinib and sorafenib have been shown to reduce proliferation, increase apoptosis, and reduce proangiogenic factor expression in vitro [128,129,130,131]. Both MKIs inhibit growth and angiogenesis in xenografts [128,129,131,132], but this effect was even greater for sunitinib if combined with rapamycin [105,133], evofosfamide [134], anti-HIFα shRNA, or topotecan [116]. Only one study using sorafenib in patients with metastatic neuroblastoma has been published in which short-term tumor stabilization was observed, but all four children finally relapsed [135].

Imatinib also increases apoptosis and reduces viability and VEGF expression [105,136,137]. This effect is enhanced if combined with doxorubicin, etoposide, or vincristine, or with the human immunodeficiency virus (HIV) protease inhibitor saquinavir [137,138]. This effect was not found when combined with irinotecan, cisplatin, or melphalan [137]. When treatment with imatinib is simultaneous with retinoic acid, inhibitory effect is also increased [139,140], but the pretreatment with retinoic acid causes cell resistance to imatinib and gamma radiation [139]. In a phase II study, no response was observed in 11 patients with neuroblastoma treated with imatinib (440 mg/m^2^/day) [141]. In another phase II study with higher imatinib doses (170 mg/m^2^ to 300 mg/m^2^ twice a day), five complete remissions (21%) and two partial responses (8%) were observed [142]. Ten years later, two of these patients had died by neuroblastoma progression and one from a second malignancy, but three of them were still alive [143].

Cabozantinib is also effective in cellular and animal models of neuroblastoma, as monotherapy or in combination with retinoic acid, topotecan, and temozolomide [144]. In patients, good results were obtained in four children with relapsed metastatic neuroblastoma, obtaining two complete remissions for more than 12 months and two stabilizations for at least 6 months [145], but studies with a larger number of patients are necessary.

Trebananib in patients with neuroblastoma is well tolerated, but the response produced in a study involving patients with several relapsed solid tumors was limited to the stabilization in one patient for 18 months [93].

As a novelty in the previously mentioned antiangiogenics, the anti-endoglin antibody TRC105 eliminates CD105+ cells in the TME in a neuroblastoma animal model, increasing the effect of immunotherapy with anti-GD2 antibody dinutuximab and activated natural killer cells [146].

## 5. Antiangiogenics and Wilms’ Tumor

Wilms’ tumor or nephroblastoma is the most common kidney cancer in childhood. It usually appears in children under five years of age as an asymptomatic mass. Five-year survival is over 90% in high-income countries and the usual treatment is nephrectomy and systemic chemotherapy [147].

High MVD is associated with poor prognosis in Wilms’ tumor [148,149]. However, Ozluk and collaborators described that vascularization does not depend on VEGF, as it presents several patterns in the different components of the tumor [148]. Other authors argue that MVD is linked to VEGF and that both are associated with worse prognosis [150,151], although high levels of VEGF-A and VEGF-C appear only in the tumor and not in the serum [152].

The treatment of Wilms’ tumor patients with bevacizumab combined with other drugs like topotecan or vincristine and irinotecan stabilized the disease or even achieved an almost complete response, but patients ended up relapsing [153,154,155]. By combining bevacizumab with irinotecan, vincristine, and temozolomide, partial remission in two patients and complete response in two other patients from one study [156] and objective response in three patients of another study [157] were observed. On the other hand, the administration of bevacizumab, sorafenib, and low doses of cyclophosphamide in children with Wilms’ tumor increases the risk of pneumothorax [158].

Sorafenib produced no response in Wilms’ tumor patients in a phase II study [159], but benefits have been reported in a few cases [160]. In a phase I study, cabozantinib showed partial response in Wilms’ tumor patients [98].

Results of the studies based on classic antiangiogenics show little benefit in Wilms’ tumor. But since high MVD is related to a poor prognosis, the study of other Wilms’ tumor proteins involved in angiogenesis, such as Wilms’ Tumor 1 Protein (WT1) [161], or c-kit [162], which is also one of the targets of imatinib, needs further research.

## 6. Antiangiogenics and Soft-Tissue Sarcomas

Soft-tissue sarcomas are a heterogeneous group of tumors that arise from mesenchymal tissues [163,164]. With current multimodal therapy (surgery, radiation, and chemotherapy), OS is around 60–70%, but it is much lower in recurrent or metastatic patients [163].

In soft-tissue sarcomas, VEGF levels correlate with tumor grade, while elevated Ang2 levels are associated with an increased tumor size [165]. However, there is no relationship between survival and VEGF concentrations, except in leiomyosarcoma, where VEGF overexpression is associated with reduced survival [166].

### 6.1. Rhabdomyosarcomas

Rhabdomyosarcomas are tumors of the skeletal muscle and constitute around 50% of soft-tissue sarcomas. The current five-year survival rate varies depending on age, tumor size, location, resectability, the presence of PAX3/PAX7-FOXO1 fusion, the condition of the nodes, and metastases [164].

Simultaneous expression of VEGF and VEGFR exists in rhabdomyosarcomas and a VEGF autocrine signaling loop is involved in cell proliferation [163,167]. The compassionate use of bevacizumab in patients with recurrent solid tumors, including several sarcomas, is safe and even generates partial responses [153]. However, the addition of bevacizumab to conventional chemotherapy for rhabdomyosarcomas does not increase PFS [168]. No objective response was observed with treatment with aflibercept, but side effects such as pain, prolonged thromboplastin time, or necrosis were produced [169].

Pazopanib is the first FDA-approved drug for metastatic soft sarcoma in adults, as it increases PFS [170,171]. In the PPTP, pazopanib was associated with PFS increase in rhabdomyosarcoma, but no objective responses were seen [170]. However, a phase I clinical trial involving sarcomas, brain tumors, and other refractory solid tumors showed that pazopanib can stabilize alveolar rhabdomyosarcoma [97], and may even produce complete remission in alveolar rhabdomyosarcoma or partial remission in embryonal rhabdomyosarcoma if combined with vincristine and irinotecan [172].

Although, according to PPTP, sorafenib reduces tumor size in xenografts, it had little activity in patients with rhabdomyosarcoma [170]. Accordingly, a phase II study found no objective response [159]. The combination of sorafenib with karineticin, cyclophosphamide, irinotecan, and temozolomide also failed to stop the progression of the tumor in a girl with metastatic alveolar rhabdomyosarcoma [173].

Other antiangiogenics have only been tested in rhabdomyosarcoma preclinical models. Sunitinib has activity in four out of six rhabdomyosarcoma cell lines of the PPTP [127], and inhibits tumor growth in xenograft models [167]. In the case of cabozantinib and imatinib, several studies show that they inhibit the growth and invasion of rhabdomyosarcoma cells [167,170,174]. Imatinib also inhibits xenograft growth [167,174], but one third of the mice create resistance [174]. Another study on the contrary argues that imatinib as single agent has no effect on rhabdomyosarcoma cells, but enhances antitumor activity of topotecan both in vitro and in vivo [175].

### 6.2. Other Soft-Tissue Sarcomas

They are a very large group of sarcomas that are more frequent in adolescents and adults. Their current treatment includes surgery, chemotherapy, and/or radiotherapy, but many of them are resistant.

Several antiangiogenics have been used in this extensive group. Positive response publication bias cannot be ruled out in the case reports. Some publications reported responses to bevacizumab in children diagnosed with desmoid-type fibromatosis [176] and metastatic alveolar soft part sarcoma (ASPS) [177,178]. Bevacizumab with gemcitabine and docetaxel produced a partial response in metastatic refractory undifferentiated sarcoma [179], and 12-month PFS in a patient with angiosarcoma [180], while combined with vincristine, irinotecan, and temozolomide stabilized the disease in liposarcoma [157]. The combination of bevacizumab with sorafenib and low doses of cyclophosphamide generates partial response in patients with synovial sarcoma and rhabdoid tumor [181].

Pazopanib showed prolonged response in patients with relapsing synovial sarcoma [182], partial response in desmoplastic small round cell tumor (DSRCT), and stabilization in other non-rhabdomyosarcomas when used as single agent [97,183]. It also reduced the size of fibrosarcoma and undifferentiated soft-tissue sarcomas, enabling its resection [184,185]. In contrast, a case of metastatic ASPS has been published in which pazopanib controlled the disease, but produced side effects [186]. In combination with other drugs, such as vincristine and irinotecan, pazopanib generates partial remission in undifferentiated sarcoma and long-term stabilization of DSRCT [172], and improves the response to chemotherapy and reduces recurrence rates in children and adults with large, unresected, intermediate- or high-grade chemosensitive soft-tissue sarcomas [187]. Another VEGFR-inhibitor, axitinib, produced a partial response in ASPS and stabilized the disease in other soft-tissue sarcomas in a phase I study [188].

Sunitinib generates response or at least stabilizes the disease in patients with ASPS [189,190]. Moreover, it is believed to penetrate in the CNS, as brain metastases from ASPS patients improved with the treatment with sunitinib [191]. Two cases of ASPS have also been described in which sunitinib was combined with radiation, allowing tumor resection in one case and producing a partial response in the other [192]. In turn, sorafenib combined with topotecan produced partial response and disease stabilization in two fibromatosis patients in a phase I study [193]. Responses to imatinib are unpredictable. It showed little or no activity in some patients [141,194], but it may be useful in desmoid tumors [195] and dermatofibrosarcoma, avoiding extensive surgery [196,197,198]. There are also promising results with cabozantinib and ARQ-197, which stabilized ASPS [98,199]. Cabozantinib also may generate a partial response in clear cell sarcoma and synovial sarcoma [98].

## 7. Antiangiogenics and Bone Sarcomas

Primary bone tumors include osteosarcoma and Ewing’s sarcoma as the most common types in childhood [200]. With current therapy, five-year survival rates are 70–80% in located forms and 30% in patients with metastases, poor response to chemotherapy, or relapses [200].

### 7.1. Osteosarcomas

Osteosarcomas normally occur at long bones and their most common symptoms are severe pain and spontaneous fractures [200].

There is controversy about the relationship between angiogenic factors and survival in osteosarcoma. Most studies argue that an increased MVD correlates with high risk of metastasis and worse prognosis [200,201,202], but others found no correlation [203], or even that they have better prognosis, longer survival, and better response to the treatment [204]. Some authors defend that VEGF overexpression correlates with poor prognosis [167,200,205,206,207], but others do not find any correlation [208]. Elevated levels of FGF [202,208] and PDGF [167,209] are also described. Therefore, several antiangiogenics have been tested in osteosarcoma. Although they are generally well tolerated, the response is variable.

Bevacizumab as a single agent does not improve survival. Problems in wound healing after surgery have also been shown, so it is advisable to cancel bevacizumab 28 days before surgery and not to resume it until 28 days later [210]. When combined with docetaxel and gemcitabine, bevacizumab generates a high response rate in osteosarcoma [211]. However, if it is combined with vincristine, irinotecan, and temozolomide [157] or with tensirolimus and sorafenib [212], it does not prevent progression and metastases. Moreover, the combination of bevacizumab, sorafenib, and low-dose cyclophosphamide increases the risk of pneumothorax, as in other cancers [158].

Axitinib stabilized refractory osteosarcoma in two children [188], whereas pazopanib, despite good preclinical results [123], did not prevent progression [213,214,215]. Among MKIs, sorafenib stabilized the disease [213,216], maintaining a prolonged partial response during 51 months in a child with refractory osteosarcoma [217]. Sunitinib was only able to prevent progression in one out of the five osteosarcoma patients [213].

One of the best clinical responses obtained with antiangiogenics in osteosarcomas was using cabozantinib. The CABONE study, which included children and adults, showed that 37% of patients had a PFS at least 33% longer than when using other conventional therapies [215]. The only study published on imatinib showed no response, although it should be noted that the dose was lower than in other studies [141,167]. In vitro, cytotoxic activity of imatinib is variable. Although it inhibits PDGF signaling, MAPK is constitutively activated, which could explain the uncontrolled cell growth and the relative unresponsiveness to imatinib [209]. The results obtained with ramucirumab in preclinical models are not very promising either. Ramucirumab inhibits the cord formation of osteosarcoma cells in vitro, but its analogue DC101 has limited efficacy in xenografts as single agent or combined with chemotherapy [122].

### 7.2. Ewing’s Sarcoma

Ewing’s sarcoma is a tumor composed of small undifferentiated round cells that normally appears in bones, but may also appear in soft tissues [200,218]. It is characterized by chromosomal translocation between EWSR1 and FLI1 genes (90% of cases), or by the fusion of EWSR1 with other transcription factors of the ETS gene family (10% of cases) [200,218].

The prognosis impact of MVD is controversial [219], probably due to the diffuse pattern in microvessel distribution [220]. There is also debate about VEGF, although most studies found elevated levels and some authors even link this overexpression to a worse prognosis [219].

The response of Ewing’s sarcoma patients to antiangiogenic therapy is also variable in clinical reports. The treatment of three patients with recurrent Ewing’s sarcoma using bevacizumab, vincristine, irinotecan, and temozolomide produced one complete remission, one partial remission, and one stabilization that finally relapsed [157,221]. The combination of bevacizumab, gemcitabine, and docetaxel did not prevent progression in another patient [179]. Axitinib was used in a patient with refractory Ewing’s sarcoma, stabilizing the disease for more than six months [188], while pazopanib maintained remission for over a year in a patient whose multiple lesions had disappeared after intensive chemotherapy [222].

Imatinib as single agent and at low doses had hardly any activity in patients with Ewing’s sarcoma [141,167]. However, it enhances the cytotoxic activity of metformin. Moreover, the addition of imatinib and metformin to the treatment with cyclophosphamide and ifosfamide increases its effectiveness both in vitro and in vivo, which could allow reducing the doses of these two drugs [223]. Imatinib also inhibits the proliferation of Ewing’s sarcoma cell line and the growth of xenografts [167,224,225], although this response is directly related to C-kit expression [226].

According to the CABONE study, treatment with cabozantinib showed a greater objective response than the other VEGFR2 inhibitors in solid tumors [227], achieving at least the stabilization of patients with Ewing’s sarcoma [98].

With regard to antiangiogenics that have only been tested in preclinical models, sunitinib has a high activity in four out of five cell lines of Ewing’s sarcoma of PPTP [127] and ramucirumab inhibits cord formation in vitro [122]. The ramucirumab analogue for in vivo studies, DC101, also generates response in xenograft models [122].

## 8. Conclusions and Future Perspectives

Most of these studies include a small number of patients or describe isolated clinical cases (Table S1). Therefore, although it seems that some antiangiogenics could be beneficial, it is hard to obtain firm conclusions. Moreover, most patients do not receive only antiangiogenic treatment, but it is combined with chemotherapy and/or radiotherapy, so it is difficult to know if the effects are produced by the angiogenic inhibitor. Furthermore, positive response publication bias cannot be ruled out in the publications.

**Figure 2:**
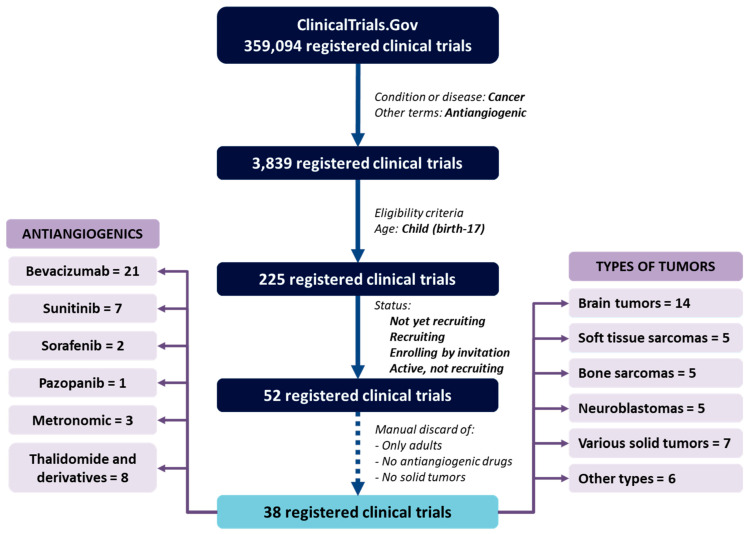
Active clinical trials that use antiangiogenic drugs in childhood cancer. The ClinicalTrial.gov database has 359,094 registered clinical trials at the time of this submission. If these trials are filtered by condition or disease (cancer) and other terms (antiangiogenic), the number is reduced to 3839. If only trials involving children (child: birth–17 years) and those that are active are selected, there are 225 clinical trials. After manually eliminating trials that include only adults, that are not solid tumors, and that do not use antiangiogenic treatments, there are 38 trials. Most of these study brain tumors, but there are also trials that include soft-tissue sarcomas, bone sarcomas, neuroblastomas, or others. Seven of the 38 trials allow the inclusion of patients with different types of cancer. In relation with antiangiogenics used, most trials use bevacizumab combined with other treatments, but there are also others directed against alternative proangiogenic pathways such as sunitinib, sorafenib, and pazopanib. Metronomic therapy, thalidomide, and its derivatives are also studied in the current clinical trials.

Research on new targets for the development of efficient antiangiogenic therapy and new clinical trials with larger numbers of patients are necessary. Currently, there are 38 clinical trials that use antiangiogenic drugs and include pediatric patients with solid tumors registered in ClinicalTrial.gov (Figure 2, Table S2), the largest database of clinical trials in the world. Most of them use bevacizumab combined with other therapeutical agents, but sunitinib, sorafenib, and pazopanib are also used. There are also pediatric clinical trials with metronomic therapy, thalidomide, or its derivatives. In relation with type of tumors, brain tumors are the most studied but it should be noted that there are seven clinical trials that include patients with very different types of cancer and whose interpretation can be difficult. It is also remarkable that many of these trials include not only pediatric patients.

Finally, it should be mentioned that the results obtained from antiangiogenic drugs in adults are disappointing, since the response of these drugs is limited and resistance appears soon [13,22]. Another disadvantage for children that must be added is the anomalies in the growth of cartilage and the potential adverse effect in the development [228]. Furthermore, it has been shown that complete inhibition of tumor angiogenesis results in hypoxia, allowing the selection of the most resistant tumor cells and the creation of an immunosuppressive TME, which inhibits the patient antitumor response [229]. However, the goal pursued with the antiangiogenics took a radical turn when Dr. Jain demonstrated that the administration of low doses, instead of depriving tumor cells of nutrition, leads to vascular normalization, increasing the perfusion and facilitating the arrival of cytotoxics, immunotherapy, and oxygen needed for some types of radiotherapy [229,230]. The benefit of the combination of antiangiogenic drugs with new therapies like inhibitors and immunotherapy for pediatric cancer needs further research in clinical trials. This is an unexplored field, but preliminary results in preclinical and adult patients are actually promising.

In summary, as one of the specific abilities of malignant tumor cells is the angiogenesis induction, research in this field and the development of new antiangiogenic drugs could contribute to improve the effectiveness of therapies against pediatric cancer and the prognosis of solid tumors, especially in neoplasias with high inter- and intratumoral heterogeneity and lack of targeted therapies.

## Supplementary Materials

The following are available online at https://www.mdpi.com/2072-6694/13/2/253/s1, Table S1: Summary of studies that include children with solid tumors treated with antiangiogenic drugs, Table S2: Clinical trials registered in ClinicalTrials.gov at the time of this submission that include pediatric patients with solid tumors and use antiangiogenic drugs.

## Abbreviations

ALL: acute lymphoblastic leukemia; Ang, angiopoietin; ASPS, alveolar soft part sarcoma; CML, chronic myeloid leukemia; CNS, central nervous system; DIPG, diffuse intrinsic pontine glioma; DSRCT, desmoplastic small round cell tumor; EC, endothelial cell; ECM, extracellular matrix; EMA, European Medicine Agency; FAK, focal adhesion kinase; FDA, Food and Drug Administration; FGF, fibroblast growth factor; FGFR, fibroblast growth factor receptor; GIST, gastrointestinal stromal tumor; HGF, hepatocyte growth factor; HIV, human immunodeficiency virus; MVD, microvascular density; PDGF, platelet-derived growth factor; PDGFR, platelet-derived growth factor receptor; PFS, progression-free survival; PPTP, pediatric preclinical testing program; OS, overall survival; TGF-β, transforming growth factor β; TME, tumor growth factor; VEGF, vascular endothelial growth factor; VEGFR, vascular endothelial growth factor receptor; WT1, Wilms’ tumor 1 protein.
