# Supplementary material for: Use of Antiangiogenic Therapies in Pediatric Solid Tumors
Source: Cancers (Basel). 2021 Jan 12;13(2):253. doi: 10.3390/cancers13020253 (PMC7827326; doi:10.3390/cancers13020253)
Supplement: Supplementary file 1 [file cancers-13-00253-s001.pdf]

Table S1. Summary of studies that include children with solid tumors treated with antiangiogenic drugs.

Tumor type		Treatment	Diagnostic	Patients	Age (years)	Best response	Worst response	Reference
Brain tumor	Low-grade glioma	BVZ	Optic nerve glioma	4	1.2-4	PR (x4)	PR (x4)	[56]
			Pilocytic astrocytoma	3	0.92-7	PR (x2)	PD (x1)	[56]
			Ganglioglioma	1	1.92	PR	PR	[56]
			Pilomyxoid astrocytoma	1	1.92	PR	PD	[68]
			Visual pathway glioma	1	2.6	PR	Side effects	[66]
			LGG	15	1-20	CR (x3)	Toxicity (x1)	[58]
			Visual pathway glioma	3	6-13	PR (x2)	PD (x1)	[60]
		BVZ+IRO	Pilocytic astrocytoma	5	3.1-11.2	PR (x5)	PR (x5)	[65]
			Pilomyxoid astrocytoma	2	7.9-12.2	PR (x2)	PR (x2)	[65]
			Oligodendroglioma	1	11.1	PD	PD	[65]
			Ganglioglioma	3	4.1-16.8	PR (x2)	SD (x1)	[65]
			Optic nerve glioma	2	9-15	PR (x1)	SD (x1)	[63]
			LGG	35	0.6-17.6	PR (x2)	PD (x8)	[61]
			Pilocytic astrocytoma	10	1.8-15.3	PR (x4)	PD (x1)	[62]
			Pleomorphic xanthoastrocytoma	1	9.4	PD	PD	[62]
			LGG	5	3.9-9.2	PR (x2)	SD (x3)	[62]
			Pilocytic astrocytoma	4	4.67-12.17	PR (x2)	PD (x3)	[57]
			Pilomyxoid astrocytoma	1	3	SD	PD	[57]
			Fibrillary astrocytoma	3	1.92-11.08	CR (x1)	PD (x3)	[57]
			Other LGG	6	1-13.42	PR (x2)	PD (x6)	[57]
			Visual pathway glioma	1	11	PR	SD	[60]
			Fibrillary astrocytoma	3	1.5-11.1	CR (x1)	SD (x1)	[64]
			Pilocytic astrocytoma	2	3.75-9.8	PR (x1)	MR (x1)	[64]
			Pilomyxoid astrocytoma	1	3	SD	SD	[64]
			Other LGG	4	3-9.6	PR (x2)	Side effects	[64]
		BVZ+TMZ	Pleomorphic xanthoastrocytoma	1	13.4	PR	PR	[153]
		BVZ+CHEM	Pilocytic astrocytoma	9	5-17	PR (x3)	PD (x3)	[59]
			Pleomorphic xanthoastrocytoma	1	18	SD	PD	[59]
			Ganglioglioma	1	18	SD	SD	[59]
			LGG	4	6-13	SD (x4)	PD (x1)	[59]
		AFLI	Pilocytic astrocytoma	1	1.9-21.6	PD	PD	[169]
		PAZ	LGG	2	3.8-23.9	PD (x2)	PD (x2)	[97]
		SORA	Pilocytic astrocytoma	6	3.5-13.8	PD (x6)	PD (x6)	[69]
			Pilomyxoid astrocytoma	2	3-9.2	PR (x1)	PD (x1)	[69]
			Ganglioglioma	1	15.1	SD	SD	[69]
			Fibrillary astrocytoma	1	6	PD	PD	[69]
			LGG	1	13.4	PD	PD	[69]
			LGG	1	6	-	Side effects	[70]
		CAB	Gliomas	6	4-18	PD (x6)	PD (x6)	[98]
		TREB	Astrocytoma	3	2.3-21	PD (x3)	PD (x3)	[93]
	Medulloblastoma	BVZ+IRO	Medulloblastoma	1	14.5	PD	PD	[65]
			Medulloblastoma	2	6-9	SD (x1)	PD (x1)	[63]
		BVZ+IRO±TMZ	Medulloblastoma	9	0-18	CR (x3)	PD (x3)	[84]

		BVZ+IRO+TMZ+VIN	Medulloblastoma	1	3.9-19.4	PR	PR	[157]
		BVZ+TEM	Medulloblastoma	2	3-14	SD (x1)	Side effects	[74]
		BVZ+CHEM	Medulloblastoma	7	7-24	CR (x5)	Death (x1)	[72]
		CAB	Medulloblastoma	1	4-18	PD	PD	[98]
		PAZ	Medulloblastoma/PNET	2	3.8-23.9	PD (x2)	PD (x2)	[97]
	High-grade glioma	BVZ+IRO	Recurrent HGG	18	5.6-20.1	SD (x8)	PD (x13)	[82]
			DIPG	17	2.9-14.6	SD (x5)	PD (x13)	[82]
			Recurrent HGG	1	7	PD	PD	[80]
			Anaplastic oligoastrocytoma	1	11.5	PR	PR	[65]
			Anaplastic oligodendroglioma	4	4.3-20.4	PR (x2)	PD (x1)	[65]
			Glioblastoma	3	6.2-11	PD (x3)	PD (x3)	[65]
			DIPG	2	4.1-8.2	PR (x1)	SD (x1)	[65]
			Grade III glioma	1	12.2	PR	PR	[65]
			DIPG	2	5	SD (x1)	PD (x1)	[63]
			Recurrent glioblastoma	1	5	CR	CR	[83]
		BVZ+IRO+TMZ	Recurrent HGG	5	5-19	SD (x2)	PD (x5)	[80]
		BVZ+IRO+TMZ+CEL+THA	Recurrent HGG	1	5	SD	SD	[80]
		BVZ+IRO+TMZ+VIN	Glioblastoma	1	18	SD	SD	[221]
		BVZ+TMZ+RT	HGG	62	3-17	OR (x12)	Death (x3)	[86]
			DIPG	2	7-11	PR (x2)	PR (x2)	[73]
		BVZ+TEM	Glioblastoma	2	3-14	PR (x1)	SD (x1)	[74]
			DIPG	1	3-14	SD (x1)	SD (x1)	[74]
		BVZ+CCNU	Recurrent HGG	1	14	PD	PD	[80]
		BVZ+DABRA+TRAME	Anaplastic pleomorphic xanthoastrocytoma	1	16	SD	Death	[67]
		BVZ+RT/BVZ+IRO	DIPG	15	3-26	Death (x15)	Death (x15)	[85]
		BVZ+RT+TMZ/BVZ+IRO+TMZ	Anaplastic astrocytoma	8	3-27	CR (x2)	Death (x3)	[85]
			Glioblastoma	4	7-29	CR (x1)	Death (x3)	[85]
		PAZ	HGG	6	3.8-23.9	PD (x6)	PD (x6)	[97]
		SORA+VAL	Glioblastoma	1	0.8	PR	PR	[88]
		SUNI	HGG	16	4.7-19.9	SD (x3)	PD (x9)	[89]
		TREB	Anaplastic astrocytoma	4	2.3-21	SD (x1)	PD (x3)	[93]
			Glioblastoma	3	2.3-21	PD (x3)	PD (x3)	[93]
			Malignant glioma	2	2.3-21	PD (x2)	PD (x2)	[93]
	Ependymoma	BVZ+IRO	Ependymoma	4	1.3-12	SD (x2)	PD (x2)	[65]
			Ependymoma	2	3-4	PR (x2)	PR (x2)	[63]
			Ependymoma	14	3-16.7	SD (x3)	PD (x1)	[94]
		BVZ+TEM	Ependymoma	1	3-14	SD (x1)	SD (x1)	[74]
		BVZ+IRO+TMZ+VIN	Ependymoma	1	10	SD	SD	[221]
		BVZ+CHEM	Ependymoma	1	7	PD	PD	[96]
		AFLI	Ependymoma	1	1.9-21.6	PD	PD	[169]
		PAZ	Ependymoma	4	3.8-23.9	SD (x1)	PD (x3)	[97]
		SUNI	Ependymoma	13	3-16.9	PR (x1)	PD (x11)	[89]
		CAB	Ependymoma	2	4-18	SD (x1)	PD (x2)	[98]
		TREB	Ependymoma	2	2.3-21	PD (x2)	PD (x2)	[93]

	Others	BVZ	PNET	1	16.0	PD	PD	[65]
		BVZ+IRO+TMZ+VIN	Atypical teratoid/rhabdoid	1	1	SD	SD	[221]
		BVZ+CHEM	Rhabdoid	3	1-6	CR (x1)	SD (x2)	[72]
			Pineoblastoma	2	12-27	CR (x1)	SD (x1)	[72]
			PNET	4	8-13	PD (x4)	Death (x4)	[72]
		BVZ+CXRT	Somatotropinoma	1	4	SD	SD	[100]
		PAZ	Atypical teratoid/rhabdoid	1	3.8-23.9	PD	PD	[97]
			Germ cell	2	3.8-23.9	PD (x2)	PD (x2)	[97]
		SORA+IRO+TMZ	Medulloephitelioma	1	3	SD	PD	[102]
		SUNI+SIRO+THA+VORI	Choroid plexus carcinoma	1	0.3	PR	PR	[103]
		TREB	PNET	1	2.3-21	PD	PD	[93]
Neuroblastoma		BVZ+IRO	Neuroblastoma	1	9	PD	PD	[63]
		BVZ+CAB+IRO+RT	Neuroblastoma	1	5.5	CR	CR	[153]
		BVZ+IRO+TMZ	Neuroblastoma	33	2.2-25.8	CR (x1)	PD (x9)	[113]
		BVZ+IRO+TMZ+VIN	Neuroblastoma	2	4-10	SD (x2)	SD (x2)	[221]
		BVZ+CHEM	Neuroblastoma	1	15.1	Death	Death	[153]
		BVZ+RTIM	Neuroblastoma	1	3.5	Side effects	Side effects	[121]
		AFLI	Neuroblastoma	2	1.9-21.6	PD	PD	[169]
		AXI	Neuroblastoma	1	5-17	PD	PD	[188]
		SORA	Neuroblastoma	4	4-5	SD (x3)	Death (x4)	[135]
		SORA+TOPO	Neuroblastoma	1	8-18	PD	PD	[193]
		IMA	Neuroblastoma	24	2-18-2	CR (5x)	PD (x9)	[142]
			Neuroblastoma	14	12-28.2	CR (x3)	Death (x10)	[143]
			Neuroblastoma	10	3-29	PD (x10)	PD (x10)	[141]
		CAB	Neuroblastoma	2	4-18	PD (x2)	PD (x2)	[98]
			Neuroblastoma	4	6-11	CR (x2)	PD (x2)	[145]
		TREB	Neuroblastoma	4	2.3-21	SD (x1)	PD	[93]
Wilms' tumor		BVZ+IRO+VIN	Wilms' tumor	2	5-10	PR (x2)	PD (x2)	[155]
		BVZ+IRO+TMZ+VIN	Wilms' tumor	3	3.9-19.4	CR (x2)	PR (x1)	[157]
			Wilms' tumor	1	11	PD	PD	[221]
			Wilms' tumor	4	7-17	CR (x2)	PD (x4)	[156]
		BVZ+SORA+CYCLO	Wilms' tumor	3	1.1-22.4	SD (X2)	PD (X1)	[181]
		BVZ+CHEM	Nephroblastoma	2	11.8-13.9	PD	Death	[153]
		AFLI	Wilms' tumor	1	1.9-21.6	PD	PD	[169]
		AXI	Nephroblastoma	1	5-17	PD	PD	[188]
		PAZ	Wilms' tumor	1	3.8-23.9	PD	PD	[97]
		CAB	Wilms' tumor	2	4-18	PR (x1)	PD (x2)	[98]
			Wilms' tumor	1	19	PR	PD	[160]
		SORA	Wilms' tumor	10	7-18	SD (x2)	PD (x8)	[159]
			Wilms' tumor	1	16	SD	PD	[160]
		ARQ-197+SIRO+SVV	Wilms' tumor	1	17.5	PD	PD	[160]

Soft-tissue sarcoma	Rhabdomyosarcoma	BVZ+IRO+TMZ+VIN	Rhabdomyosarcoma	1	12	PD	PD	[221]
Soft-tissue sarcoma	Rhabdomyosarcoma	BVZ+CHEM	Alveolar rhabdomyosarcoma	1	14.6	Death	Death	[153]
Soft-tissue sarcoma	Rhabdomyosarcoma	AFLI	Alveolar rhabdomyosarcoma	1	1.9-21.6	PD	PD	[169]
Soft-tissue sarcoma	Rhabdomyosarcoma	AXI	Alveolar rhabdomyosarcoma	1	5-17	PD	PD	[188]
Soft-tissue sarcoma	Rhabdomyosarcoma	PAZ	Rhabdomyosarcoma	5	3.8-23.9	SD (x1)	PD	[97]
Soft-tissue sarcoma	Rhabdomyosarcoma	PAZ+IRO+VIN	Alveolar rhabdomyosarcoma	5	5-19	CR (x1)	PD (x2)	[172]
Soft-tissue sarcoma	Rhabdomyosarcoma	PAZ+IRO+VIN	Embryonal rhabdomyosarcoma	3	5-19	PR (x1)	SD (x2)	[172]
Soft-tissue sarcoma	Rhabdomyosarcoma	CAB	Embryonal rhabdomyosarcoma	2	4-18	PD (x2)	PD (x2)	[98]
Soft-tissue sarcoma	Rhabdomyosarcoma	SORA	Rhabdomyosarcoma	10	5-21	PD (x10)	PD (x10)	[159]
Soft-tissue sarcoma	Rhabdomyosarcoma	SORA+TOPO	Embryonal rhabdomyosarcoma	1	8-18	PD	PD	[193]
Soft-tissue sarcoma	Rhabdomyosarcoma	SORA+KARI+CYCLO+IRO+TMZ+RT	Alveolar rhabdomyosarcoma	1	8	PD	Death	[173]
Soft-tissue sarcoma	Rhabdomyosarcoma	TREB	Alveolar rhabdomyosarcoma	1	2.3-21	PD	PD	[93]
Soft-tissue sarcoma	Rhabdomyosarcoma	TREB	Embryonal rhabdomyosarcoma	4	2.3-21	PD (x4)	PD (x4)	[93]
Soft-tissue sarcoma	Non-rhabdomyosarcoma	BVZ	Desmoid-type fibromatosis	1	16	PR	PR	[176]
Soft-tissue sarcoma	Non-rhabdomyosarcoma	BVZ+IFN	ASPS	1	8	PR	PR	[177]
Soft-tissue sarcoma	Non-rhabdomyosarcoma	BVZ+IRO+TMZ+VIN	Liposarcoma	1	3.9-19.4	SD	SD	[157]
Soft-tissue sarcoma	Non-rhabdomyosarcoma	BVZ+IRO+TMZ+VIN	Synovial sarcoma	1	3.9-19.4	SD	Toxicity	[157]
Soft-tissue sarcoma	Non-rhabdomyosarcoma	BVZ+IRO+TMZ+VIN	Angiosarcoma	1	3.9-19.4	PD	PD	[157]
Soft-tissue sarcoma	Non-rhabdomyosarcoma	BVZ+IRO+TMZ+VIN	Clear cell sarcoma	1	16	PD	PD	[221]
Soft-tissue sarcoma	Non-rhabdomyosarcoma	BVZ+SORA+CYCLO	Synovial sarcoma	3	1.1-22.4	PR (x2)	PD (x1)	[181]
Soft-tissue sarcoma	Non-rhabdomyosarcoma	BVZ+CEL	ASPS	1	5	PR	PD	[178]
Soft-tissue sarcoma	Non-rhabdomyosarcoma	BVZ+CED	ASPS	2	1.5-30	SD (x1)	PD (x2)	[192]
Soft-tissue sarcoma	Non-rhabdomyosarcoma	BVZ+CHEM	Undifferentiated sarcoma	1	15	PR	PD	[179]
Soft-tissue sarcoma	Non-rhabdomyosarcoma	BVZ+CHEM	Angiosarcoma	1	4.4	PR	PD	[180]
Soft-tissue sarcoma	Non-rhabdomyosarcoma	AFLI	Synovial sarcoma	1	1.9-21.6	PD	PD	[169]
Soft-tissue sarcoma	Non-rhabdomyosarcoma	AFLI	Other sarcomas	4	1.9-21,6	SD (x1)	PD (x3)	[169]
Soft-tissue sarcoma	Non-rhabdomyosarcoma	AXI	ASPS	2	5-17	PR (x1)	PD (x2)	[188]
Soft-tissue sarcoma	Non-rhabdomyosarcoma	AXI	Epitheliod sarcoma	1	5-17	PD	PD	[188]
Soft-tissue sarcoma	Non-rhabdomyosarcoma	AXI	Malignant peripheral nerve sheat tumor	2	5-17	SD (x1)	PD (x1)	[188]
Soft-tissue sarcoma	Non-rhabdomyosarcoma	AXI	ASPS	1	17	PD	PD	[211]
Soft-tissue sarcoma	Non-rhabdomyosarcoma	PAZ	Synovial sarcoma	4	3.8-23.9	SD (x1)	PD (x3)	[97]
Soft-tissue sarcoma	Non-rhabdomyosarcoma	PAZ	ASPS	3	3.8-23.9	SD (x2)	PD (x1)	[97]
Soft-tissue sarcoma	Non-rhabdomyosarcoma	PAZ	DSRCT	2	3.8-23.9	CR (x1)	PD (x1)	[97]
Soft-tissue sarcoma	Non-rhabdomyosarcoma	PAZ	Clear cell sarcoma	2	3.8-23.9	PD (x2)	PD (x2)	[97]
Soft-tissue sarcoma	Non-rhabdomyosarcoma	PAZ	Other sarcomas	5	3.8-23.9	SD (x2)	PD (x3)	[97]
Soft-tissue sarcoma	Non-rhabdomyosarcoma	PAZ	DSRCT	29	6.3-50.1	CR (x1)	PD (x11)	[183]
Soft-tissue sarcoma	Non-rhabdomyosarcoma	PAZ	ASPS	1	11	PR	PD	[186]
Soft-tissue sarcoma	Non-rhabdomyosarcoma	PAZ	ASPS	2	12-17	SD (x2)	PD (x2)	[211]

			Synovial sarcoma	2	13-14	PR (x2)	PD (x2)	[182]
			Fibrosarcoma	1	0.33	PR	PR	[184]
			ASPS	1	1.5-30	SD	PD	[192]
		PAZ+IRO+VIN	Undifferentiated sarcoma	1	5-19	PD	PD	[172]
			Clear cell sarcoma	1	5-19	SD	SD	[172]
			DSRCT	1	5-19	SD	SD	[172]
			Other sarcomas	1	5-19	PD	PD	[172]
		PAZ+IFOS+CAR+ETO	Soft tissue sarcoma	1	0.25	PR	PR	[185]
		PAZ+EVE	ASPS	1	1.5-30	PR	PD	[192]
		SORA	ASPS	2	12-17	PR (x1)	PD (x1)	[211]
			Angiosarcoma	1	4.2	PD	PD	[180]
			ASPS	1	1.5-30	PD	PD	[192]
		SORA+TOPO	Fibromatosis	2	8-18	PR (x1)	PD (x1)	[193]
		SUNI	ASPS	4	2-21	PR (x2)	Side effects (x1)	[190]
			ASPS	2	12-17	PR (x1)	PD (x2)	[211]
			ASPS	2	8-13	PR (x2)	PR (x2)	[189]
			ASPS	1	1.5-30	PR	PD	[192]
		SUNI+RT	ASPS	2	1.5-30	PR (x2)	PR (x2)	[192]
		IMA	Fibromatosis	51	12-67	SD (x43)	PD (x5)	[195]
			ASPS	2	1.5-30	SD (x1)	Death (x2)	[192]
			DSRCT	8	9-32	SD (x1)	Death (x1)	[194]
			DSRCT	10	3-29	PD (x10)	PD (x10)	[141]
			GIST	1	3-29	PD	PD	[141]
			Synovial sarcoma	4	3-29	PD (x4)	PD (x4)	[141]
			Dermatofibrosarcoma protuberans	1	1-12	PR	PR	[196]
			Dermatofibrosarcoma protuberans	1	1.5	PR	PR	[197]
			Dermatofibrosarcoma protuberans	3	3-14	CR (x2)	PR (x3)	[198]
		CAB	ASPS	2	4-18	PD (x2)	PD (x2)	[98]
			Synovial sarcoma	1	4-18	Toxicity	Toxicity	[98]
			Clear cell sarcoma	1	4-18	PR	PD	[98]
			ASPS	1	17	PD	Side effects	[211]
		Foretinib	ASPS	2	1.5-30	SD (x1)	PD (x2)	[192]
		ARQ-197	ASPS	2	12-15	SD (x2)	SD (x2)	[199]
			ASPS	1	1.5-30	SD	PD	[192]
		TREB	ASPS	1	2.3-21	PD	PD	[93]
			Synovial sarcoma	1	2.3-21	PD	PD	[93]
		TREB+CHEM	ASPS	1	1.5-30	PD	Death	[192]
Bone sarcoma	Osteosarcoma	BVZ+IRO+TMZ+VIN	Osteosarcoma	2	3.9-19.4	SD (x1)	PD (x2)	[157]
		BVZ+SORA+CYCLO	Osteosarcoma	3	1.1-22.4	SD (X3)	SD (X3)	[181]
		BVZ+SORA+TEM	Osteosarcoma	1	16	PD	PD	[212]
		BVZ+CHEM	Osteosarcoma	31	6.8-20.3	PR (x4)	PD (x6)	[210]
			Osteosarcoma	8	15-27	CR (x3)	PD (x1)	[211]
		AXI	Osteosarcoma	2	5-17	SD (x2)	SD (x2)	[188]
		PAZ	Osteosarcoma	4	3.8-23.9	SD (x1)	PD (x3)	[97]
			Osteosarcoma	3	12-16	SD (x3)	PD (x3)	[214]
		SORA	Osteosarcoma	8	6.7-19.9	PR (x6)	SD (x2)	[216]

			Osteosarcoma	1	7	PR	SD	[217]
		SORA+TOPO	Osteosarcoma	4	8-18	PD (x4)	PD (x4)	[193]
		IMA	Osteosarcoma	10	3-29	PD (x10)	PD (x10)	[141]
		CAB	Osteosarcoma	2	4-18	PD (x2)	PD (x2)	[98]
		TREB	Osteosarcoma	4	2.3-21	PD (x4)	PD (x4)	[93]
	Ewing's sarcoma	BVZ+IRO+TMZ+VIN	Ewing's sarcoma	1	3.9-19.4	SD	PD	[157]
			Ewing's sarcoma	2	20-22	CR (x1)	PR (x1)	[221]
		BVZ+CHEM	Ewing's sarcoma	2	13-19	PR	SD	[179]
		AFLI	Ewing's sarcoma	1	1.9-21.6	PD	PD	[169]
		AXI	Ewing's sarcoma	3	5-17	SD (x1)	PD (x3)	[188]
		PAZ	Ewing's sarcoma	3	3.8-13.9	PD (x3)	PD (x3)	[97]
		PAZ+IRO+VIN	Ewing's sarcoma	5	5-19	PR (x1)	PD (x1)	[172]
		PAZ+TOPO+MEL+CYCLO	Ewing's sarcoma	1	14	PR	PR	[222]
		SORA	Ewing's sarcoma	2	2.6-18.5	PR (x1)	PD (x1)	[216]
		SORA+TOPO	Ewing's sarcoma	3	8-18	PD (x3)	PD (x3)	[193]
		IMA	Ewing's sarcoma	24	3-29	PR (x1)	PD (x23)	[141]
		CAB	Ewing's sarcoma	4	4-18	SD (x1)	PD (x3)	[98]
		TREB	Ewing's sarcoma	3	2.3-21	PD (x3)	PD (x3)	[93]

Abreviations: AFLI, Aflibercept; ARQ-197, Tivantinib; AXI, Axitinib; BVZ, Bevacizumab; CAB, Cabozantinib; CAR, Carboplatino; CCNU, Carmustine; CED, Cediranib; CEL, Celedoxib; CHEM, Chemotherapy; CR, Complete response; CXRT, Chemo-radiotherapy; ETO, Etoposide; EVE, Everolimus; IFN, Interferon alpha-2B; IFOS, Ifosfamide; IMA, Imatinib; IRO, Irinotecan; KARI , Karinethicin; MEL, Melphalan; MR, Minor response; OR, Objective response; PAZ, Pazopanib; PD, Progressive disease; PR, Partial response; RT, Radiation; RTIM, Radioimmunotherapy; SD, Stable disease; SIRO, Sirolimus; SORA, Sorafenib; SUNI, Sunitinib; SVV, Seneca Valley virus; TEM, Temsirolimus; THA, Thalidomide; TMZ, Temozolomide; TREB, Trebananib; VAL, Valproic acid; VIN, Vincristine; VORI, Vorinostat.

Table S2. Clinical trials registered in ClinicalTrials.gov at the time of this submission that include pediatric patients with solid tumors and use antiangiogenic drugs.

Status	ClinicalTrials.gov identifier	Phase	Intervention	Condition/Tumor type	Eligible ages	Starting date	Estimated completation date
Not yet recruiting	NCT02897986	1	Metronomic vinorelbine Propranolol	Various refractory/relapsing solid tumors	4-21 years	January 2017	January 2021
	NCT03554707	1	Bevacizumab Irinotecan Temozolomide Radiation Genetic (SGT-53)	Recurrent/progressive/refractory brain tumors	1-21 years	June 2021	December 2023
	NCT04607421	3	Bevacizumab Encorafenib Cetuximab Oxaliplatin Irinotecan Leucovorin 5-FU Capecitabine	Colorectal cancer with BRAF V600E mutation	16-older	November 2020	November 2026

Recruiting	NCT01661400	1	Metronomic cyclophosphamide Thalidomide	Various solid tumors	6 months-21 years	October 2012	November 2022
	NCT02446431	Early 1	Bevacizumab Cyclophosphamide Valproic acid Temsirolimus	Soft tissue sarcoma and bone sarcoma	12 months-31 years	July 2014	July 2029
	NCT03063983	2	Metronomic methotrexate Cyclophosphamide	Osteosarcoma	16-30 years	January 2017	January 2022
	NCT01356290	2	Bevacizumab Thalidomide Celecoxib Fenofibric acid Etoposide Cyclophosphamide Etoposide phosphate Cytarabine	Various recurrent brain tumors	0-19 years	April 2014	April 2023
	NCT02698254	N/A	Bevacizumab Radiation	Various recurrent brain tumors	All ages	July 2016	July 2020
	NCT01552434	1	Bevacizumab Cetuximab Tensirolimus Valproic acid	Various refractory/relapsing solid tumors	All ages	March 2020	March 2021
	NCT03686254	2/3	Bevacizumab Second-line chemotherapy	Colorectal cancer	16-80 years	July 2018	October 2023
	NCT02693535	2	Sunitinib Crizotinib Palbociclib Temsirolimus Transtuzumab+Pertuzumab Vemurafenib+Cobimetinib Regorafenib Olaparib Pembrolizumab Nivolumab+Ipilimumab Abemaciclib Afatinib Talazoparib	Advanced solid tumors, lymphoma non-Hodgkin and multiple mieloma	12 years-older	March 2016	December 2024
	NCT02840409	2	Bevacizumab Vinblastine	Low grade glioma	6 months-18 years	August 2016	August 2026
	NCT03277924	1/2	Sunitinib Nivolumab	Soft tissue sarcoma and bone sarcoma	12-80 years	May 2017	September 2022
	NCT03900793	1	Sunitinib Losartan	Osteosarcoma	10-40 years	August 2019	February 2025
	NCT04049864	Early 1	Lenalidomide DNA vaccine Salmonella vaccine	Relapsed neuroblastoma	1-20 years	January 2019	December 2021
	NCT01837862	1/2	Bevacizumab Mebendazole Vincristine Carboplatin	Various brain tumors	1-21 years	October 2013	April 2020

			Temozolomide<br>Irinotecan				
	NCT01884740	1/2	Bevacizumab<br>SIACI of Erbitux	Various brain tumors	1-21 years	June 2013	January 2025
	NCT02573896	1	Lenalidomide<br>Ch14.18<br>NK cells	Neuroblastoma	1 month-30 years	November 2018	August 2022
	NCT02308527	2	Bevacizumab<br>Temozolomide<br>Irinotecan<br>Topotecan<br>Dinutuximab beta<br>Cyclophosphamide	Neuroblastoma	1-21 years	July 2013	July 2026
	NCT02303028	1/2	Pazopanib<br>Topotecan	Relapsed/refractory solid tumors	2-21 years	March 2015	December 2020
	NCT01189643	Early 1	Bevacizumab<br>Irinotecan<br>Temozolomide<br>High dose alkylator	DSRCT	1-29 years	August 2010	August 2021
	NCT03257631	2	Pomalidomide	Various brain tumors	1-21 years	August 2017	March 2023
	NCT01187199	1	Bevacizumab<br>Sorafenib<br>Temsirolimus<br>Paclitaxel<br>Carboplatin	Relapsed/refractory advanced o metastatic cancer	All ages	August 2010	December 2021
	NCT01015222	1	Bevacizumab<br>Dasatinib<br>Paclitaxel	Relapsed/refractory advanced o metastatic cancer	All ages	November 2009	November 2021
	NCT00329043	2	Sunitinib<br>LHRH agonist	Prostate cancer	All ages	May 2006	December 2021
Active, not recruiting	NCT01946529	2	Bevacizumab<br>Sorafenib<br>Vincristine<br>Doxorrubicin<br>Cyclophosphamide<br>Ifosfamide<br>Etoposide<br>Temozolomide<br>Tensirolimus<br>Radiation	DSRCT and Ewing sarcoma	0-25 years	December 2013	July 2026
	NCT02415153	1	Pomalidomide	Recurrent/refractory brain tumors	3-20 years	July 2015	July 2019
	NCT02115074	1	Celebrex (COX-2 inhibitor)<br>Fluvastatine	Glioma	6-21 years	June 2014	October 2022
	NCT01396408	2	Sunitinib<br>Temsirolimus	Advanced rare tumors	16 years-older	July 2011	December 2020
	NCT00569127	3	Bevacizumab<br>Octreotide acetate<br>Recombinant IFNα-2b	Various carcinoid and neuroendocrine tumors	All ages	December 2007	January 2015
	NCT01217437	2	Bevacizumab<br>Irinotecan<br>Temozolomide	Various recurrent brain tumors	0-21 years	November 2010	December 2017
	NCT01236560	3	Bevacizumab<br>Temozolomide<br>Vorinostat	High-grade glioma	3-21 years	November 2010	September 2016

	NCT01391962	2	Sunitinib Cediranib	Alveolar soft part sarcoma	16 years-older	July 2011	December 2021
	NCT01553149	2	Lenalidomide	Recurrent brain tumors	0-21 years	March 2012	June 2020
	NCT01711554	1	Lenalidomide Dinutuximab Isotretinoin	Recurrent/refractory neuroblastoma	0-21 years	February 2013	September 2018
	NCT00715442	2	Sunitinib	Renal Cell Carcinoma	All ages	June 2008	June 2020
	NCT01767792	2	Bevacizumab	Neurofibromatosis type 2 and progressive vestibular Schwannomas	6 years-older	May 2013	September 2020
	NCT02017717	3	Bevacizumab Nivolumab Ipilimumab	Recurrent glioblastoma	All ages	January 2014	April 2021
